# Vertebral compression fractures in pediatric patients with intestinal failure: A prospective observational case series.

**DOI:** 10.1016/j.intf.2024.100006

**Published:** 2024-07-16

**Authors:** Asia Smith, Shweta S. Namjoshi, Laura K. Bachrach, Monica Grover, Christine Hoyer, James CY Dunn, Mark R. Hanudel, Sivan Kinberg

**Affiliations:** a Howard University School of Medicine, USA; b Division of Pediatric Gastroenterology, Hepatology and Nutrition, Stanford University School of Medicine, USA; c Division of Endocrinology, Stanford University School of Medicine, USA; d Division of Pediatric Gastroenterology, Hepatology and Nutrition, Columbia University Irving Medical Center, USA; e Division of Pediatric Surgery, Department of Surgery, Stanford University School of Medicine, USA; f Division of Pediatric Nephrology, David Geffen School of Medicine UCLA, USA

**Keywords:** Bone health, Fracture, Pediatric, Short bowel syndrome, Intestinal failure, Vertebral fracture

## Abstract

**Background:**

Intestinal failure associated bone disease (IFABD) poses a significant threat to bone health in pediatric patients with intestinal failure (IF) reliant on life-saving parenteral nutrition (PN).

**Methods:**

This observational study sought to determine the prevalence of vertebral fractures (VF) detected through lateral thoracolumbar x-rays using Genant criteria in pediatric patients with IF at a single center. It was conducted as part of a larger observational database study of intestinal failure long term outcomes.

**Results:**

Fourteen out of 79 patients with IF were included in the study. The median age of our cohort was 9 years. Short bowel syndrome (SBS) was the cause of IF in nine patients (64 %), with two patients each having a pediatric onset congenital diarrhea & enteropathy (14 %) or dysmotility (14 %) as their primary IF diagnosis, and one patient (7 %) with normal intestinal length and absorption, requiring PN for growth support in the context of chronic vomiting. Three of 14 participants (21 %) had vertebral fractures confirmed on x-rays, two of whom were asymptomatic. Notably, bisphosphonate therapy was initiated in one patient with VF, highlighting the clinical impact of early detection.

**Conclusion:**

Despite study limitations, including a small single center sample size, the identification of VF in 3 out of the 14 participants underscores the need for further investigation in pediatric patients with IF, especially because 2 of these 3 patients were asymptomatic. Multicenter studies in the future may explore risk factors for VF to create guidelines for radiographic screening, surveillance, and treatment in this pediatric population.

## Introduction

Pediatric intestinal failure (IF) is defined as a reduction of gut mass below what is needed to facilitate adequate nutrition and growth, resulting in a dependence on supplemental parenteral support for a minimum of 60 days [1]. Short bowel syndrome (SBS) is the most common cause of IF in children, which typically occurs after surgical bowel resection, secondary to congenital or perinatal diseases such as necrotizing enterocolitis (NEC), bowel atresia, gastroschisis, and volvulus.

Intestinal failure associated bone disease (IFABD) is a known and common complication of IF [1]. While consensus criteria for IFABD have not been established, osteopenia and osteoporosis have been commonly observed complications of IF over the decades during which parenteral nutrition (PN) has been a life-saving therapy. The occurrence of a low-trauma bone fracture suggests IFABD and has been observed in children as young as 18 months. [2], [3], [4], [5], [6] As such, there is a critical need to identify the risk factors for fracture in children with IF undergoing intestinal rehabilitation as well as the most appropriate age and time frame for screening for early intervention.

The pathophysiology of IFABD is complex, multifactorial, and not fully understood. Bone mineralization using intravenous minerals in PN may be yield less effective mineralization than with enteral calcium and phosphorus intake, and there is limited guidance on optimization of parenteral calcium or phosphorus delivery after infancy. [7], [8], [9] Important minerals and micronutrients for bone mineralization (calcium, phosphorus, magnesium, copper, vitamin D, vitamin K) can be malabsorbed, especially when there is fast transit due to ultra short bowel syndrome or fat malabsorption due to small bowel resection. [10] Co-morbid conditions in IF, such as immobility due to gross motor delay and insufficient weight bearing exercise reduce the biomechanical stimulation needed for optimal bone development. Chronic inflammation associated with inflammatory bowel disease (IBD), chronic liver disease (CLD), chronic kidney disease (CKD), and perhaps small bowel bacterial overgrowth (SBBO) may negatively impact mineral absorption and contribute to bone loss. [11], [12], [13], [14] Metabolic acidosis from dehydration and loss of bicarbonate in stool and medications such as steroids, loop diuretics, proton pump inhibitors, seizure medications and low molecular weight heparin [15] may contribute to poor bone health. [16] Lastly, complex relationships among phosphate homeostasis, fibroblast growth factor 23 (FGF23), iron deficiency, and CKD, and how these interactions affect bone health, are currently being investigated. [17], [18] Insufficient accrual of bone mass especially in childhood can lead to chronic osteopenia and osteoporosis. [6] Children with IF are at risk for all of the comorbidities associated with poor bone health, and IFABD and fracture are unfortunately common in this population.

Options for screening of IFABD include Dual-energy x-ray absorptiometry (DXA), which evaluates bone density, and plain film radiography, which screens for vertebral fractures. DXA has robust normative data and minimal radiation exposure. [19] However, DXA has limitations because it provides a measure of two-dimensional bone mineral density that must be adjusted for delays in growth and puberty, which are common in IF. DXA scans in children must be interpreted using pediatric reference standards (and not adult) to avoid misinterpretation of values. Technical limitations, such as difficulty remaining still during the scan, can lead to motion artifact and potentially inaccurate results. In addition, low trauma fractures can occur despite normal DXA results, especially in patients receiving glucocorticoid therapy, as bone quality declines faster than quantity. [20].

While there are no strict criteria for IFABD, in 2013, a panel of pediatric bone experts from the International Society of Clinical Densitometry (ISCD) revised the diagnostic criteria for pediatric osteoporosis to include: the presence of one or more vertebral compression fractures (VF) occurring without major trauma or local disease, or the presence of low bone mineral content or density for age (Z-score <−2.0) plus a clinically significant fracture history of long bone fractures (≥ two fractures by age 10 years OR ≥ three by age 19 years). [21], [22] VF can be identified by a lateral thoracolumbar x-ray using the Genant criteria to identify changes in the morphology of individual vertebrae. [22].

The advantage of Genant’s method [23] is that it provides information regarding the severity of a vertebral fracture, assessing the fracture based on visual estimation of vertebral height reduction and morphological change. Each vertebra receives a severity grade based on the degree of vertebral height loss, ranging from grade 0 (normal) to grade 3 (severe fracture). This approach is well-suited as a foundation for standardized interpretation of vertebral factures for several reasons: it is faster than morphometric methods that involve measuring all vertebral dimensions [24], it surpasses nonstandardized qualitative assessments in accuracy [25], it demonstrates high reproducibility [26], and it does not require a referral outside of clinic, especially if the clinic has radiology embedded in the clinic. Additionally, an estimated 40 % of patients with VF do not complain of back or bone pain, making the x-ray an important screening study for pediatric patients at high risk for osteoporosis. [27], [28] If VFs occur without major trauma, the patient meets criteria for osteoporosis whether bone density is determined to be low by DXA or not. Treatment with a bisphosphonate can be indicated on a compassionate use basis. [29] For these reasons, our center uses Genant films for screening for IFABD instead of DXA.

Detection of the full spectrum of IFABD from osteopenia to fracture requires increased knowledge of the prevalence of asymptomatic fractures in patients with IF and determination of feasible screening methods given a high pre-test probability of osteoporosis in this population. The aim of this study was to determine the prevalence of VF detected on lateral thoracolumbar x-rays using Genant criteria in pediatric patients with IF.

## Methods

Between September 1st 2020 and December 1st 2021, we conducted a prospective observation of VF detected on screening lateral thoracolumbar x-rays (Genant films) in pediatric patients with IF. As most patients with intestinal failure are at risk for IFABD, radiographs were obtained as part of routine care for patients generally age 5 and older at a single institution for Intestinal Rehabilitation & Nutrition Support (IR & NS). Follow-up imaging was only obtained if fracture or osteopenia was identified on films. The medical record was reviewed to explore clinical risk factors for poor bone health. Inclusion criteria included any patient in the IR & NS program who had completed a lateral thoracolumbar radiograph to rule out VF using Genant criteria during the observation period. Exclusion criteria included solid organ transplant recipient status, patients with oncologic diagnoses, and any patient not cared for in the IR & NS program continuously for more than 6 months.

Institutional Review Board approval and informed consent for assessment of long-term outcomes in IF was obtained. Data was stored securely in the Research Electronic Data Capture (REDCap) database. This study was conducted as part of a larger effort to characterize current and past outcomes of patients followed by our institution’s IR & NS program. The demographics, diagnoses, types of nutrition therapy, surgical procedures, venous access data, central line infection rate data, and medication use data were recorded securely in this database. Lateral spine x-rays were interpreted using Genant criteria, as reviewed by radiologists at our institution. [22].

## Results

A total of 14 out of 79 patients in our IR & NS program were included in this study. Patient demographics for the cohort of fourteen patients are described in Table 1. The median age of the population was nine years, with an interquartile range (IQR) of eight to thirteen years of age, and a maximum age of nineteen years old. There were eight male patients and six female patients. No patients nor parents identified the patients as transgender nor non-binary, and none were on hormonal therapy or puberty blockers for gender affirmation during the study period to our knowledge. The racial distribution of the cohort consisted of 43 % Latino, 36 % White, 14 % Black, and 7 % Asian. Short bowel syndrome (SBS) was the cause of IF in nine patients (64 %), with two patients each having a pediatric onset congenital diarrhea & enteropathy (14 %) or dysmotility (14 %) as their primary IF diagnosis, and one patient (7 %) with normal intestinal length and absorption who required PN for growth support in the context of chronic vomiting. One patient had congenital bone disease as part of their cause of IF due to a biallelic pathogenic variant in *UNC45A,* and one patient had Ehlers Danlos Syndrome (EDS) with a genetic variant not known to impact bone fragility. Eight patients used enteral feeding for less than 25 % of their total estimated calories for age, with 75 % clinician estimated PN dependency, and 3 patients were 50 % PN dependent. Among the 3 patients who met approximately 75 % of their needs enterally, one patient was hypometabolic due to ischemic brain injury, and was nearly 100 % dependent on PN for fluids and electrolytes but not at all dependent on PN for calories. One patient achieved enteral autonomy after the study period and was actively weaning lipids and fluids during the study period, and one patient was on PN two days per week for minimal fluid and calorie support.

**Table 1:** Demographics of patients with intestinal failure and radiographs of the lateral thoracolumbar spine ordered between September 1st, 2020 and December 1st, 2021.

		Number of Patients (%)	Median (IQR)
Age	0 to 2 years* 3-4 years 5-10 years 11-17 years 18 years or older	1 (7) 0 (0) 6 (42) 5 (36) 2 (14)	2 (2,5)
Self-Identified or Parent Identified Gender	Male Female Transgender on hormone therapy Transgender without hormone therapy Non-binary	8 (57) 6 (43) 0 0 0	
Self-Identified Race/Ethnicity	Asian or Pacific Islander Black Latino Other Indigenous SWANA White	1 (7) 2 (14) 6 (43) 0 0 0 5 (36)	
Primary Diagnosis	Short bowel syndrome Pediatric Onset Congenital Diarrhea & Enteropathy Dysmotility Other	9 (64) 2 (14) 2 (14) 1 (7)	
Estimated percentage enteral feeding	< 25 % 50 % 75 % 100 %	8 (57) 3 (21) 3 (21) 0	

* *Film obtained at age 18 months for severe scoliosis occurring in the context of cerebral palsy and immobility.

* SWANA Southwest Asian & North African

Table 2 provides clinical details for the VF observed during the study period. Three patients (21 %) had VF identified on screening lateral spinal films. One patient (participant #7) had VF and vertebral pain symptoms and received bisphosphonate therapy. This patient was a teenage person with EDS. The second patient with VF (participant #3) was a 9-year-old male who was asymptomatic. The third patient with VF (participant #6) was a 10-year-old male with end-stage liver disease was lost to follow up and did not receive bisphosphonate therapy. Two out of fourteen patients (14.3 %) had a history of low-trauma femur fractures but did not have VF. Among these two patients, one was an 8-year-old male with congenital bone disease (participant #12). The other (participant #4) was a chronically immobile male patient who experienced a three year long hospitalization and secondary gross motor delay. He had a history of low-trauma femur fracture during the study period. While the spinal film that was obtained during the study period did not show VF, a spinal film obtained 2 years after the study period did show VF at that time. Participant #3′s mobility improved after discharge from the hospital to a group home setting. Because participant #12 had osteopenia on plain films and a history of multiple low-trauma fractures including a femur fracture, this patient received bisphosphonate therapy though he did not have VF. Because bisphosphonate infusions can cause electrolyte abnormalities, they required admission to ensure appropriate serum calcium and phosphorus concentration after infusion. Home PN was adjusted in this patient to maintain normal serum calcium and phosphorus concentration upon discharge from the hospital.

**Table 2:** Frequency of vertebral fracture diagnosed by radiologist in patients with intestinal failure and bone health risk factors.

Subject	History Femur Fracture	Back Pain Symptoms	Radiologist Diagnosis of Vertebral Fracture	Intestinal Failure Fracture Risk Score	Qualitative Diarrhea Severity Score	Abnormal corrected serum calcium within 1 month of film*	Abnormal non-septic serum phosphorus within 1 month of film* *	Congenital Bone Disease	Decision to use bisphosphonate therapy?
1	no	no	no	7	8	no	no	no	no
2	no	no	no	12	16	no	**yes, low**	no	no
3	no	no	**Grade 1 T8 (mild)** **Grade 1 T9 (mild)**	5	16	no	no	no	no
4	**yes**	no	no	17	18	no	no	no	no
5	no	no	no	7	10	no	no	no	no
6	no	no	**yes**	13	16	no	no	no	no
7	no	**yes**	**Grade 1 T5 (mild)** **Grade 1 T7 (mild)** **Grade 1 L1 (mild)**	10	16	no	no	no	**yes**
8	no	no	no	7	16	no	no	no	no
9	no	no	no	2	0	no	no	no	no
10	no	no	no	11	16	no	no	no	no
11	no	no	no	9	0	no	no	no	no
12	**yes**	no	no	17	14	no	no	**yes**	**yes**
13	no	no	no	6	10	no	no	no	no
14	no	no	no	25	20	no	no	no	no
Total Number Positive (%) or Median	2 (14)	0	3 (21)	9.5	16	0	1(7)	1 (14)	2 (14)

* * nonseptic serum phosphorus was obtained to avoid confounding during sepsis

* serum calcium corrected for albumin was used to avoid misinterpretation when albumin was low

## Discussion

In this study of fourteen pediatric patients with IF receiving PN, we detected VF using Genant criteria in three patients. Fractures were asymptomatic in two of the three patients with VF. All three patients were developmentally able to communicate body pain. The presence of VF occurring without trauma or local disease is sufficient to diagnose pediatric osteoporosis. An additional two subjects had a history of low trauma femur fracture, indicative of abnormal bone fragility.

The finding of VF led to early initiation of bisphosphonate therapy in one patient with IFABD. Bisphosphonate therapy was also begun in one subject with a history of femur fracture in consultation with pediatric endocrinology. A third patient with VF did not receive therapy because they were lost to follow up.

We recognize several limitations of this study. This study occurred during the covid-19 pandemic between 2020 and 2021, and radiographs were only obtained in patients willing to come to clinic in person during the pandemic to complete the radiograph, and in patients who needed routine care at a frequency that may allow for screening. As such, there may be selection bias for patients with more severe IF who are being seen more frequently, or who needed to be seen in person despite the pandemic. Additional limitations were that Genant x-rays were routinely performed only in patients over the age of 5 years and only in those who remained on PN for part of their nutrition, potentially missing VF in younger children or in children who were weaned off PN. In addition, there was incomplete information in the medical records of all subjects documenting Tanner pubertal staging, aluminum concentrations, SBBO, enteral micronutrient intake, stool malabsorptive studies, and urine calcium, urine protein, and urine oxalate levels. Weight and BMI Z-scores were not included Table 1 however, no patients were chronically malnourished during the study period. These limitations reflect the reality of a busy intestinal rehabilitation clinic practice and the challenge in ordering films and obtaining results within the study time frame. In our IR & NS program, we do not routinely obtain DXA scans because we adjust bisphosphonate therapy using Genant films, and as such we screen patients with spine x-rays alone. Therefore, we do not have data on how DXA scans compare to Genant films in this paper as some centers might [30], [31], [32] and this is a limitation of our study. Lastly, while we listed demographic factors for the patients who did obtain films, we do not have dynamic year by year demographic data for the clinic population as a whole during the study period.

Despite these limitations, the finding of VF in 3 out of 14 of subjects with IF in this study and one additional patient with VF two years after the study period was completed (who initially screened negative) warrants further investigation. Vertebral fractures occurring without major trauma are an indication of abnormal bone quality and meet criteria for pediatric osteoporosis. [21], [22] There is currently no guidance for clinicians on the best age or best modality to perform a bone health screening in IF, nor is there guidance on the best interval to follow up diagnostics or repeated screening for this chronic complex disease. There is also no current risk factor-based approach to bone health screening to guide assessment in this heterogenous population.

Based upon these preliminary findings, our center continues to obtain routine lateral thoracolumbar x-rays to screen for IFABD and to follow treatment impact once bisphosphonate therapy has been initiated. The risk for VF in this population would ideally be explored through multicenter research, perhaps though a learning health systems approach. [33] If an increased risk for VF is confirmed in multicenter studies, next steps could include investigation of clinical correlations of fracture risk and the development of guidelines for radiographic screening and surveillance and use of bisphosphonates or other pharmacologic (preferably anabolic) agents for this population.

## Ethics approval

IRB approval obtained through Stanford University School of Medicine.

## Funding

None.

## CRediT authorship contribution statement

**Mark R Hanudel:** Writing – review & editing. **Sivan Kinberg:** Writing – review & editing, Writing – original draft, Formal analysis, Data curation, Conceptualization. **Asia Smith:** Writing – review & editing, Formal analysis. **Shweta S. Namjoshi:** Writing – review & editing, Writing – original draft, Formal analysis, Data curation, Conceptualization. **Laura K Bachrach:** Writing – review & editing, Writing – original draft, Formal analysis, Data curation, Conceptualization. **Monica Grover:** Writing – review & editing, Writing – original draft, Data curation, Conceptualization. **Christine Hoyer:** Writing – review & editing. **James CY Dunn:** Writing – review & editing, Data curation.

## Declaration of Competing Interest

The authors declare that they have no known competing financial interests or personal relationships that could have appeared to influence the work reported in this paper.

## Data Availability

Available upon reasonable request.
